# Successful Repair of Superior Mesenteric Artery Aneurysm with Reconstruction of Branches

**DOI:** 10.3400/avd.cr.20-00108

**Published:** 2020-12-25

**Authors:** Ryota Matsumoto, Takashi Shibuya, Fumiyoshi Saijo, Kenichi Watanabe, Yoshiki Sawa

**Affiliations:** 1 Department of Cardiovascular Surgery, Osaka University Graduate School of Medicine, Suita, Osaka, Japan

**Keywords:** superior mesenteric artery aneurysm, visceral artery aneurysm, surgical repair

## Abstract

Superior mesenteric artery aneurysms (SMAAs) are rare and potentially life-threatening. Whether surgical or endovascular repair is performed, mesenteric ischemic complication is the greatest concern. A 56-year-old gentleman with SMAA underwent surgical resection with reconstruction of the superior mesenteric artery (SMA) and its branches using the great saphenous vein with several techniques, including island reconstruction of the branches, staged segmental cross-clamping, and an external shunt, to reduce the mesenteric ischemia time. The postoperative course was uneventful with no signs of mesenteric ischemia. A computed tomography scan showed that all grafts to the SMA and its branches were patent.

## Introduction

Superior mesenteric artery aneurysms (SMAAs) are rare entities, accounting for 6.7%–6.9% of all visceral artery aneurysms (VAAs).^1,2)^ SMAAs are often symptomatic with abdominal pain; however, due to the increased use of modern imaging techniques, such as computed tomography (CT) scan and magnetic resonance imaging, more asymptomatic SMAAs have been incidentally detected. Whether symptomatic or asymptomatic, SMAAs are potentially life-threatening with a risk of rupture and embolism. Rupture of SMAA causes critical intraperitoneal hemorrhage; according to a recent study, the rupture rate at presentation was 38%.^1)^ Embolism can also lead to a lethal condition due to mesenteric infarction. Therapeutic strategies for SMAAs include surgical or endovascular interventions; however, the optimal treatment is unknown because of their rarity. The complication of mesenteric ischemia leading to mesenteric or pancreatic infarction is the most critical concern. Therefore, careful monitoring for mesenteric ischemia is required when performing either surgical or endovascular interventions for the superior mesenteric artery (SMA) and its branches. Herein, we present a case of successful surgical resection of SMAA with reconstruction of the SMA and its branches using the great saphenous vein (GSV) with several techniques such as island reconstruction with a double-barrel graft, staged segmental cross-clamping, and an external shunt, to reduce the mesenteric ischemia time.

## Case Report

The patient was a 56-year-old male with a history of hypertension and dyslipidemia, in whom a CT scan performed 9 years earlier as part of a medical checkup had detected a 15-mm-diameter SMAA. He was referred to our institution, where it was found that the aneurysm had grown to 25 mm in diameter.

**Figure 1:**
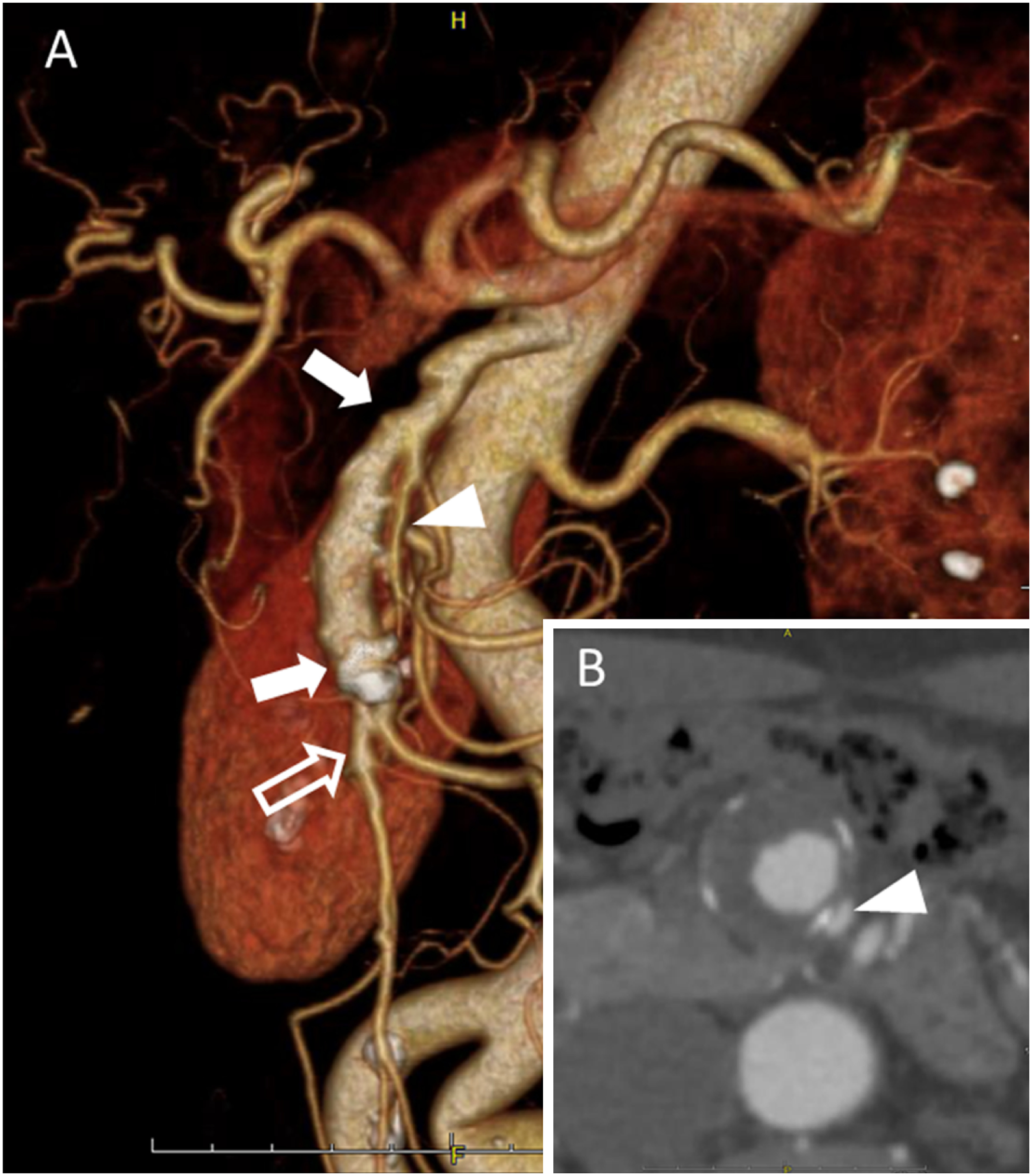
Fig. 1 Preoperative computed tomography angiography (**A**, a volume-rendered image; **B**, an axial view). The superior mesenteric artery aneurysm had a diameter of 25 mm and was located 20 mm distal to the origin of the superior mesenteric artery. The aneurysm and dissection longitudinally extended 3 cm distal to the origin of the aneurysm (➡). The true lumen of the aneurysm (▼) gave rise to middle colic artery, three jejunal arteries, and right colic artery from the proximal end. The ileocolic artery (⇒) originated from the superior mesenteric artery just distal to the end of the aneurysm.

The SMAA was located 20 mm distal to the origin of the SMA and was dissected with a patent false lumen. The aneurysm and dissection longitudinally extended 3 cm distal to the origin of the aneurysm. The true lumen of the aneurysm gave rise to the middle colic artery, three jejunal arteries (JAs), and right colic artery (RCA) from the proximal end. The ileocolic artery (ICA) originated from the SMA immediately distal to the end of the SMAA (**Fig. 1**).

Although the SMAA was asymptomatic, intervention was indicated due to its gradual expansion to a diameter of greater than 2 cm with potential risks of rupture and embolism. Endovascular treatment (EVT) with placement of a low-profile stent graft (SG) was considered to be too difficult owing to the short distal landing zone and the risk of embolism due to the intra-aneurysmal thrombus. The coverage of the branch arteries was also a concern with EVT. Therefore, we decided to perform surgical resection.

**Figure 2:**
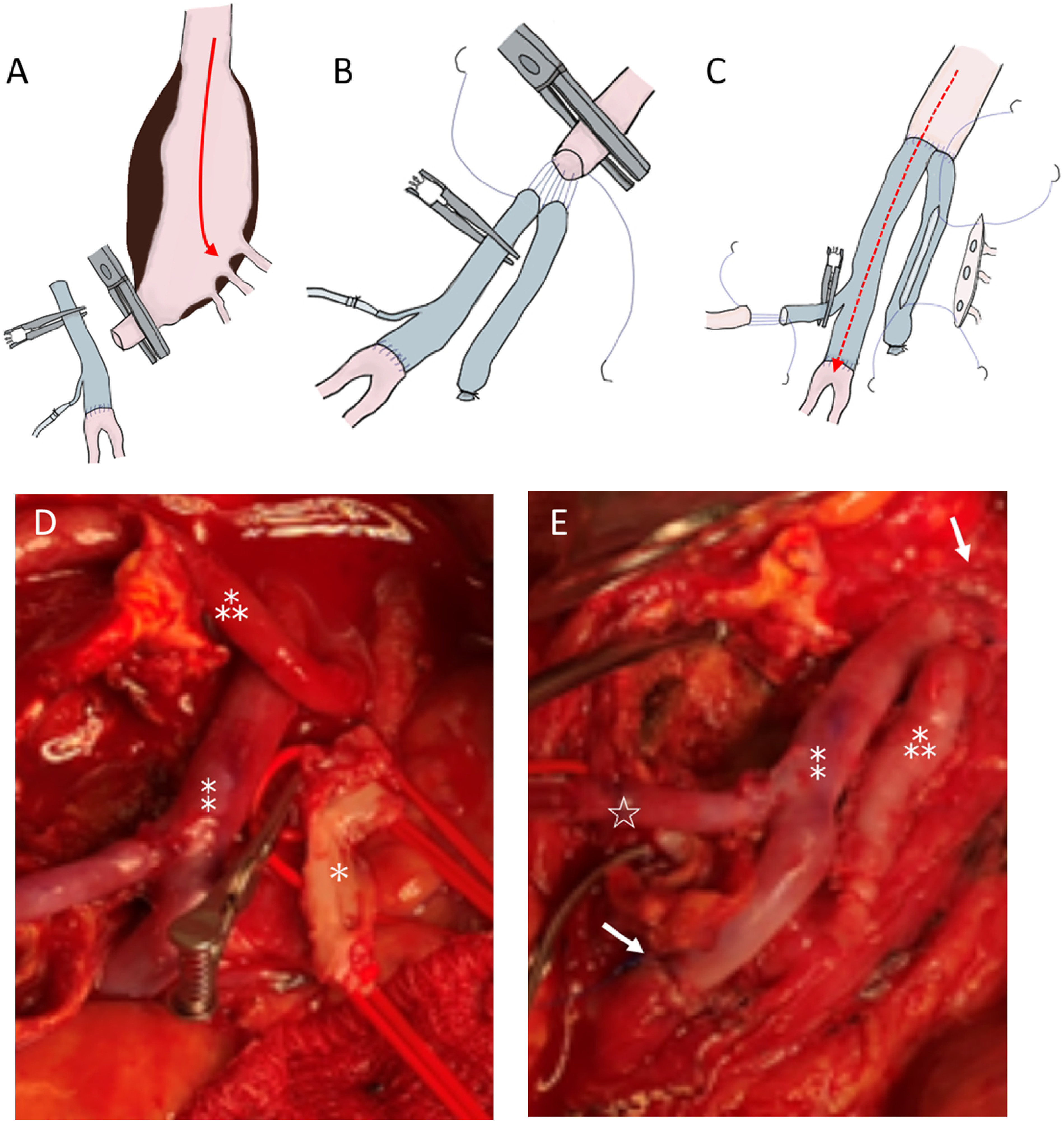
Fig. 2 Arrows describing the blood flow. (**A**) After clamping the distal portion of the superior mesenteric artery aneurysm, an end-to-end anastomosis was created between the distal end of the superior mesenteric artery and the great saphenous vein. (**B**) After resection of the aneurysm, the double-barrel graft was anastomosed to the proximal end of the superior mesenteric artery. An external shunt kept perfusion to the superior mesenteric artery and the ileocolic artery. (**C**) The three jejunal arteries were reconstructed with island technique. The right colic artery was anastomosed to the branch of the great saphenous vein. (**D**) An operative photograph of island reconstruction of the jejunal arteries (*). (**E**) An operative photograph after reconstruction. The proximal and distal ends of the anastomosis are shown with white arrows. The right leg (⁑) was anastomosed to the trunk of the superior mesenteric artery, and the left leg (⁂) was anastomosed to the jejunal arteries using the island technique. The right colic artery (☆) was anastomosed to the branch of the great saphenous vein.

The operative technique used was as follows:

1. We performed preoperative ultrasound evaluation to mark the GSV and its branches at the left groin and then harvested the GSV, preserving one major branch near the saphenofemoral junction.
2. The proximal and distal aneurysm necks were dissected and encircled through a median laparotomy. The three JAs, RCA, and ICA were also dissected.
3. After systemic heparinization, the distal portion of the SMAA was clamped while maintaining antegrade blood flow to the JAs, and an end-to-end anastomosis was fashioned between the distal end of the SMA and the GSV. A vessel cannula was introduced into the branch of the GSV and connected to a 4-Fr arterial sheath inserted into the right femoral artery to initiate distal perfusion to the SMA and ICA via the distal anastomosis. The external shunt flow was 40 ml/min. The ischemia time for the SMA and ICA was 10 min (**Fig. 2A**).
4. The proximal necks of the SMA, RCA, and JAs were clamped, and the aneurysm was opened. The intimal flap was resected, and the false lumen was opened. There was a caliber discrepancy between the proximal end of the SMA (8 mm) and GSV (4 mm); therefore, another GSV was anastomosed in a side-to-side fashion, and a double-barrel graft of the GSV was prepared, which was anastomosed to the proximal end of the SMA. The SMA and ICA were antegradely perfused from the right leg, and the external shunt was completed (**Fig. 2B**).
5. As expected from the preoperative CT angiography, none of the three JAs were involved in the dissection and arose from the true lumen. The orifices of the three JAs were trimmed en bloc (the island technique) and anastomosed to the left leg of the double-barrel graft in a side-to-end fashion. The ischemia time for the JAs was 32 min (**Fig. 2C**).
6. The RCA was reconstructed with an end-to-end anastomosis to the branch of the GSV, which had been used for the external shunt. The ischemia time for the RCA was 45 min (**Fig. 2D**).
7. The respective flow rates in the SMA, ICA, and RCA were 125, 42, and 24 ml/min.

**Figure 3:**
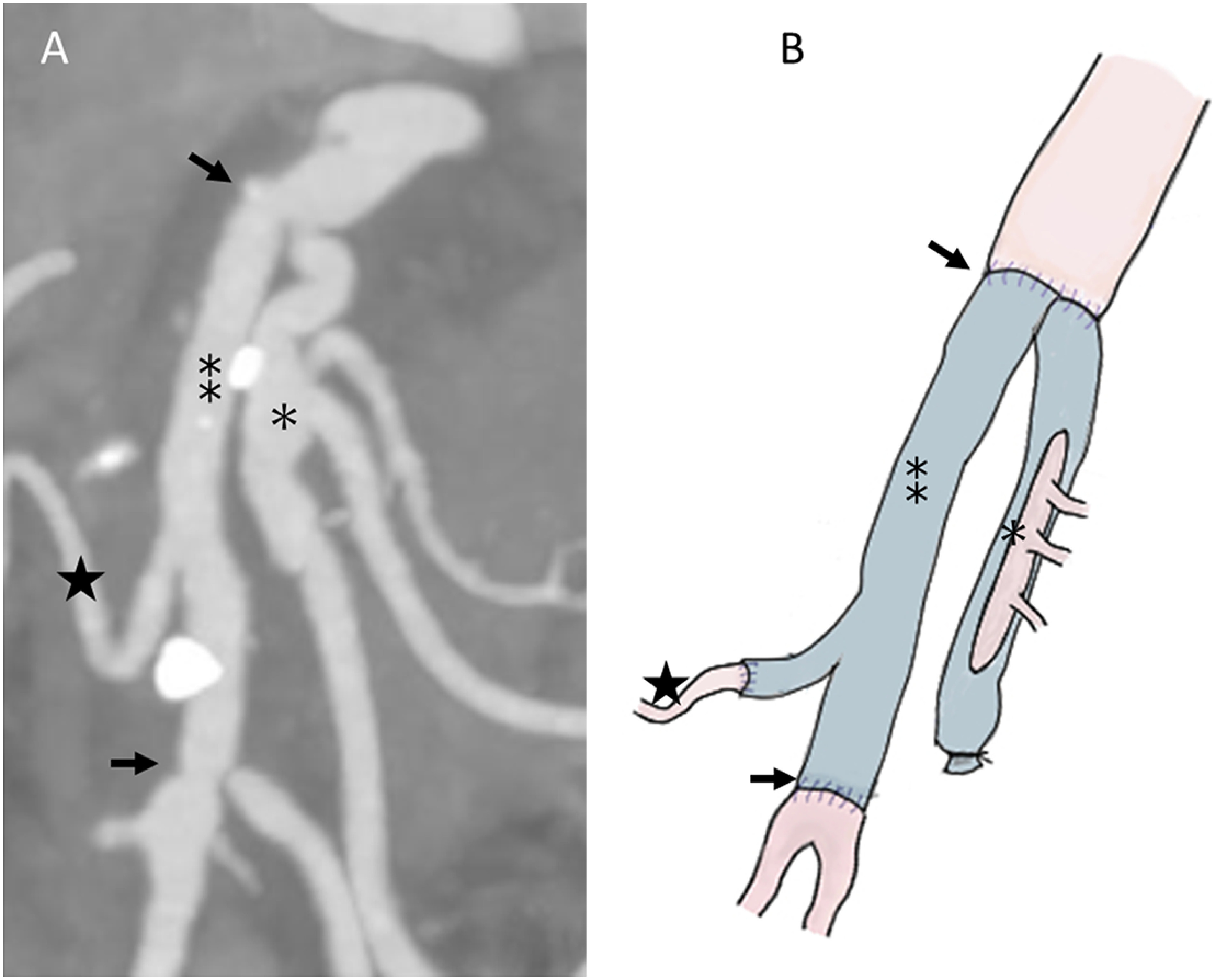
Fig. 3 (**A**) Postoperative computed tomography angiography (maximum intensity projection) and (**B**) a schematic presentation. The maximum intensity projection image confirmed complete resection of the superior mesenteric artery aneurysm and patency of all grafts, including the superior mesenteric artery and branches. * The left leg of the double-barrel graft was anastomosed to the jejunal arteries. ⁑ The right leg of the double-barrel graft. ★ Right colic artery. ➡ The proximal and distal ends of the anastomosis.

The operating time was 258 min. The patient was extubated in the operating room. The postoperative course was uneventful with no signs of mesenteric ischemia or other adverse events. The laboratory data showed temporary elevation of the amylase level on postoperative day 1, which returned to normal on the following day. In addition, aspartate aminotransferase, alanine aminotransferase, lactate dehydrogenase and creatine kinese levels were all within normal ranges. The postoperative CT scan showed complete resection of the SMAA and patency of all grafts and the SMA with its branches (**Fig. 3**). The patient was discharged on postoperative day 9. The follow-up CT scan 6 months after the surgery showed that the grafts were patent with no recurrence of SMAA.

## Discussion

SMAA are rare; however, with the development of imaging technology, an increasing number of cases have been detected.^2)^ Given the high incidence and mortality of ruptured SMAAs, they should be immediately treated upon diagnosis, regardless of the size.^1,3)^ In the present case, the etiology of aneurysmal change was likely to be associated with dissection with unclear onset. The presence of SMA dissection does not indicate repair in itself, but the aneurysmal change required intervention. The treatment options for SMAA include surgical treatment or EVT. The optimal treatment is unknown, and individual treatment is decided upon based on the location of the aneurysm and the patient’s clinical status and comorbidities.

Conventionally, SMAAs have been treated by a surgical approach. Surgical techniques include simple ligation, aneurysmorrhaphy, and resection of the aneurysm with or without revascularization. In the surgical treatment of SMAA, ischemic complications are a concern. Stone et al. reported that simple ligation could be performed safely without mesenteric ischemia.^1)^ However, mesenteric perfusion after ligation depends on collateral circulation from the celiac and inferior mesenteric arteries, and ischemic complications may lead to a lethal event, such as mesenteric or pancreatic infarction. Therefore, revascularization of the SMA may be required in some cases. Revascularization can be performed with bypass or transposition of the SMA.^4)^ Bypass techniques are classified as in situ and extra-anatomic.^5)^ In situ bypass is physiologically desirable, and kinking can be avoided by a shorter bypass, unlike extra-anatomic bypass, which retrogradely perfuses the SMA from the abdominal aorta or iliac arteries. In situ bypass is suitable for SMAA with a proximal neck of sufficient length to perform clamping and anastomosis, as in our patient. However, when the SMAA arises near the origin of artery, dissecting the proximal neck may be more difficult. In particular, dissection near the origin of the SMA can impair the nerve plexus around this vessel, leading to severe diarrhea. Various graft materials have been used, including autologous veins or arteries and prosthetic conduits (Dacron or expanded polytetrafluoroethylene grafts). The GSV is the most widely used graft material in bypass grafting surgery because surgeons can harvest a long graft and form a conduit as required for grafting with venovenostomy. We created a double-barrel graft by performing venovenostomy with a side-to-side anastomosis between two lengths of the GSV, which enabled us to address the caliber discrepancy and maintain perfusion to the SMA and ICA during reconstruction of the JAs. The limitation of this technique is that the durability of a double-barrel graft is unknown.

There is controversy over whether or not the branches of the SMA should be revascularized, with few reports mentioning branch reconstruction. However, Jiang et al. reported on a patient who underwent bypass only to the trunk of the SMA and developed an infarction in the small intestine^6)^; this implies that some patients may require revascularization of the branches as well as the trunk of the SMA. Preoperative and intraoperative evaluation of mesenteric perfusion is important. There is also the question of which branches should be revascularized, particularly in a patient with a large SMAA involving several branches. The island technique was useful for reducing the mesenteric ischemia time when multiple branches were simultaneously reconstructed. Rigorous evaluation of anatomy by preoperative CT angiography was helpful for planning the graft design.

In addition to island reconstruction, we performed several techniques to reduce the mesenteric ischemia time. One was the staged segmental cross-clamp technique, which is used to maintain perfusion distal to the clamp site when repairing a thoracoabdominal aortic aneurysm. The other technique used was an external shunt from the femoral artery to the SMA and ICA via a branch of the GSV, which is used in coronary artery bypass grafting to maintain distal perfusion.^7)^ A combination of these techniques reduced the mesenteric ischemia time, resulting in an uneventful postoperative course.

Currently, EVT for SMAA is a valid therapeutic alternative with a high success rate and low procedural morbidity and mortality. EVT can be performed with placement of an SG and/or embolization with coils, glue, or plugs. Anatomical indications for SG placement include the presence of adequate proximal and distal landing zones without excessive tortuosity.^8)^ The advantage of SG placement is its ability to exclude aneurysms while preserving blood flow in the affected vessels, thereby avoiding the risk of distal ischemic complications. However, the issue with SG placement is the coverage of major branches, which arise from aneurysms and landing zones; sacrifice of these branches can lead to mesenteric ischemia and significant morbidity if collateral circulation is insufficient. Stent thrombosis is the most critical complication after SG placement, which may cause mesenteric infarction. Some reports have described stent thrombosis that led to bowel necrosis after SG placement to the SMA.^9,10)^ Our patient had a short distal landing zone and many branches involved in the aneurysm, which would be sacrificed by SG placement. For these anatomical reasons, we elected to perform open repair.

## Conclusion

We have reported a case of successful surgical resection of a SMAA with reconstruction of the SMA and its branches using the GSV. A combination of several techniques, including island reconstruction with a double-barrel graft, staged segmental cross-clamping, and an external shunt, reduced the mesenteric ischemia time and led to an uneventful postoperative course without any complications of mesenteric ischemia.
